# Preparation and Properties of Walnut Protein Isolate–Whey Protein Isolate Nanoparticles Stabilizing High Internal Phase Pickering Emulsions

**DOI:** 10.3390/foods13152389

**Published:** 2024-07-28

**Authors:** Yanling Lu, Yuxin Jiang, Jiongna Liu, Xiaoqin Yang, Yueliang Zhao, Fangyu Fan

**Affiliations:** 1 College of Biological and Food Engineering, Southwest Forestry University, Kunming 650224, China; luyanling20220222@163.com (Y.L.); jyx0807@outlook.com (Y.J.); ljn9812@swfu.edu.cn (J.L.); 2 Key Laboratory of Forest Disaster Warning and Control of Yunnan Province, Kunming 650224, China; yangxiaoqin@swfu.edu.cn; 3 Key Laboratory of National Forestry and Grassland Administration on Biodiversity Conservation in Southwest China, Southwest Forestry University, Kunming 650224, China; 4 School of Public Health, Shanghai Jiao Tong University School of Medicine, Shanghai 200240, China; ylzhao1@sjtu.edu.cn

**Keywords:** walnut protein isolate, pH cycling, high internal phase Pickering emulsions, stability

## Abstract

To enhance the functional properties of walnut protein isolate (WalPI), hydrophilic whey protein isolate (WPI) was selected to formulate WalPI-WPI nanoparticles (nano-WalPI-WPI) via a pH cycling technique. These nano-WalPI-WPI particles were subsequently employed to stabilize high internal phase Pickering emulsions (HIPEs). By adjusting the mass ratio of WalPI to WPI from 9:1 to 1:1, the resultant nano-WalPI-WPI exhibited sizes ranging from 70.98 to 124.57 nm, with a polydispersity index of less than 0.326. When the mass ratio of WalPI to WPI was 7:3, there were significant enhancements in various functional properties: the solubility, denaturation peak temperature, emulsifying activity index, and emulsifying stability index increased by 6.09 times, 0.54 °C, 318.94 m^2^/g, and 552.95 min, respectively, and the surface hydrophobicity decreased by 59.23%, compared with that of WalPI nanoparticles (nano-WalPI), with the best overall performance. The nano-WalPI-WPI were held together by hydrophobic interactions, hydrogen bonding, and electrostatic forces, which preserved the intact primary structure and improved resistance to structural changes during the neutralization process. The HIPEs stabilized by nano-WalPI-WPI exhibited an average droplet size of less than 30 μm, with droplets uniformly dispersed and maintaining an intact spherical structure, demonstrating superior storage stability. All HIPEs exhibited pseudoplastic behavior with good thixotropic properties. This study provides a theoretical foundation for enhancing the functional properties of hydrophobic proteins and introduces a novel approach for constructing emulsion systems stabilized by composite proteins as emulsifiers.

## 1. Introduction

Emulsion is a dispersed system formed from two immiscible phases (usually an oil phase and an aqueous phase) through the input of external energy (e.g., stirring, homogenizing, dispersing, and sonication). Conventional emulsions utilize small-molecule surfactants to reduce the oil–water interfacial tension for dynamic stabilization [1]. However, conventional emulsions tend to become unstable over time, and the toxic side effects of surfactants on the human body, as well as their environmental pollution, limit their application in the food field [2]. Currently, organic food ingredients that have undergone canonization are commonly used in emulsion preparation, particularly Pickering emulsions, which have shown promising research results. Pickering emulsions are emulsions that use solid particles instead of surfactants [3]. Food-grade materials (e.g., proteins and polysaccharides), especially nanoparticles, are suitable for preparing stable Pickering emulsions due to their significant advantages, including natural origin, non-toxicity, biodegradability, excellent nutritional value, acceptability in the food industry, good surface activity, and excellent emulsion-stabilizing capabilities [4]. Among these emulsions, high internal phase Pickering emulsions (HIPEs) have received widespread attention from researchers. HIPEs are a type of highly concentrated emulsion stabilized by solid particles, with a dispersed phase volume fraction exceeding 74%. Compared to Pickering emulsions, HIPEs exhibit higher resistance to phase separation, Ostwald ripening, and agglomeration, demonstrating exceptional stability under various environmental conditions [5], such as temperature [6], centrifugation [7], wider pH, and varying ionic strengths [8].

Proteins, (e.g., zein [9], casein [10], and rice protein [11]) with their advantages of edibility, safety, and biocompatibility, are ideal materials for stabilizing HIPEs [12]. However, the emulsification effect of raw protein is not ideal, and it is necessary to modify and compound it to achieve the desired results [13]. For example, the compact structure of pea protein [14], the low solubility of coconut globulins [15], and the strong hydrophobicity of zein [16], are factors that affect their emulsification properties. Currently, physical methods, chemical processes, enzymatic modification, or their synergistic treatments are common techniques employed to overcome the limitations in protein application. Research has indicated that whey protein isolate (WPI)–almond gum nanoparticles, prepared by the ionic cross-linking method, can enhance the emulsion stability [17]. Additionally, the storage stability of microgel nanoparticle emulsions from soy protein hydrogels is improved by adding microbial transglutaminase [18]. However, traditional modification methods are limited due to the safety hazards of chemical reagents, low efficiency, and long reaction times. The pH cycling technique has become a current research hotspot for protein modification due to its easy operation, high safety, and low cost.

The pH cycling technique dissolves hydrophobic proteins with hydrophilic substances under alkaline conditions and then changes the pH to neutral. This process results in the hydrophobic groups of the proteins being buried within the structure, while the hydrophilic groups are exposed [19]. This creates a stable protein complex and simultaneously enhances the properties of the hydrophobic proteins. Researchers have used pH cycling to modify complex proteins and have achieved notable results. For example, rice protein and cod protein were combined by pH cycling to form hydrophilic co-assemblies, increasing the solubility of rice proteins to more than 90% (*w*/*v*) [20]. Additionally, hydrophobic wheat glutenin and cordyceps formed a homogeneous hydrophilic structure during pH cycling through hydrophobic interactions, with cordyceps enhancing the rigid structure of the protein [21]. These results suggest that pH cycling can enhance protein solubility without disrupting protein structure. This method is easy to operate, safe, and cost-effective, and has become a focal point in current protein modification research.

Walnut residues, a by-product of walnut oil processing, are rich in protein and represent a high-quality plant protein resource. Currently, the comprehensive utilization rate of walnut residues is low, resulting in a significant waste of protein resources [22]. Walnut protein isolate (WalPI) contains 18 amino acids, including all 8 essential amino acids, meeting the standards set by the Food and Agriculture Organization (FAO) of the United Nations and the World Health Organization (WHO) [23]. The main component of WalPI is gluten (72.06%) [24], and the high gluten content leads to poor solubility of WalPI, restricting its application in food production. Therefore, fully developing the protein resources of walnut residues and realizing their high-value utilization is of great significance in improving the comprehensive utilization of walnut residues [25]. Hydrophilic WPI has excellent solubility over a wide pH range and shows great potential in interacting with hydrophobic substances to enhance their solubility [26]. Wei et al. [27] prepared WPI-zein nanoparticles and found that the hydrophilicity and stability of composite nanoparticles were enhanced compared to WPI or zein nanoparticles alone. Kristensen et al. [28] found that the addition of WPI could promote an increase in the solubility of pea protein.

Given the promising potential of the pH cycling technique in protein modification, we utilized this method to prepare WalPI-WPI nanoparticles (nano-WalPI-WPI) with varying mass ratios, aiming to enhance the functional properties of WalPI. Specifically, we characterized the primary structure, particle size, polydispersity index (PDI), zeta potential, and interactions of the nano-WalPI-WPI. Additionally, we investigated the solubility, surface hydrophobicity (H_0_), thermal stability, and emulsifying properties of nano-WalPI-WPI. Subsequently, the impact of different nano-WalPI-WPI mass ratios on the droplet size, microstructure, storage stability, and rheological properties of HIPEs was systematically analyzed. The findings of this study offer theoretical insights for the preparation and application of protein-based HIPEs.

## 2. Materials and Methods

### 2.1. Materials

Walnut residues, corn oil, and WPI (93% protein, *w*/*w*) were purchased from Qiaojia County Local Flavor Food Co., Ltd. (Zhaotong, China), Yihai Kerry Food Industry Co., Ltd. (Kunming, China), and Shanghai Jianglai Biotechnology Co., Ltd. (Shanghai, China), respectively. The sodium dodecyl sulfate-polyacrylamide gel electrophoresis (SDS-PAGE) Gel Preparation Kit and maker protein were bought from Biomed Biotechnology Co., Ltd. (Beijing, China) and Vazyme Biotech Co., Ltd. (Nanjing, China). Coomassie Blue Fast Staining Solution was purchased from Shanghai Epizyme Biomedical Technology Co., Ltd. (Shanghai, China). All other experimental materials were analytically graded.

### 2.2. Preparation of WalPI

WalPI preparation was carried out using the alkali dissolution–acid precipitation technique, with minor adjustments, as outlined by Huang et al. [29]. The protein, water, ash, and fat contents for the WalPI were 80.15%, 8.94%, 2.94%, and 1.72%, respectively.

### 2.3. Preparation of Nano-WalPI-WPI

The nano-WalPI-WPI preparation method of Wang et al. [30] was adopted with slight modifications. The ratios of WalPI and WPI were 9:1, 8:2, 7:3, 6:4, and 1:1 (*w*/*w*), respectively, and then dissolved in distilled water to prepare protein solutions (1.0%, *w*/*v*). The solution was adjusted to pH 12.0 with NaOH (2.0 mol/L) and stirred at 500 rpm for 4 h at room temperature. Next, the solution was adjusted to neutral with HCl (2.0 mol/L), then centrifuged at 8000 rpm (Eppendorf Centrifuge 5804 R, Hamburg, Germany) for 10 min at room temperature to remove insoluble material. The supernatant was subjected to dialysis and lyophilization to obtain nano-WalPI-WPI. With the same method, individual WalPI nanoparticles (nano-WalPI) and WPI nanoparticles (nano-WPI) were prepared.

In this study, nano-WalPI-WPI with different mass ratios were named WalPI-WPI 9:1, WalPI-WPI 8:2, WalPI-WPI 7:3, WalPI-WPI 6:4, and WalPI-WPI 1:1, respectively.

### 2.4. Characteristics of Nano-WalPI-WPI

#### 2.4.1. SDS-PAGE

The method of Huang et al. [29] was referenced to determine the SDS-PAGE of nano-WalPI, nano-WPI, and nano-WalPI-WPI and slightly modified. The protein solution (0.2%, *w*/*v*) was centrifuged at 3000 rpm for 1 min at room temperature. The supernatant (40 μL) and loading buffer (10 μL) were mixed and heated in a boiling water bath for 5 min. Then, 10 μL of the sample and 5 μL of maker protein were added to the electrophoresis gel wells. The initial voltage of the electrophoresis (Bio-Rad Laboratories, Hercules, CA, USA) was set at 80 V and adjusted to 120 V after the sample entered the separation gel, continuing until the end of electrophoresis. The gel was stained using Coomassie Blue Fast Staining Solution for 1 h, and the electrophoretic bands were obtained by decolorizing with distilled water overnight.

#### 2.4.2. Solubility

The solubility of nano-WalPI, nano-WPI, and nano-WalPI-WPI was determined according to the method of Hu et al. [31] with slight modifications. The protein solution, at a concentration of 0.01% (*w*/*v*), was subjected to centrifugation at 8000 rpm for 10 min at room temperature to obtain the supernatant. Bovine serum albumin (BSA) served as the standard, and the protein content was quantified using the Coomassie Brilliant Blue assay. The protein solubility was subsequently calculated according to Formula (1).

(1) $$\mathrm{Solubility}(\%)=\frac{\mathrm{Weigh}\;\mathrm{of}\;\mathrm{protein}\;\mathrm{in}\;\mathrm{the}\;\mathrm{supernatant}\;}{\mathrm{Weigh}\;\mathrm{of}\;\mathrm{total}\;\mathrm{protein}}\times 100$$

#### 2.4.3. Particle Size, PDI, and Zeta Potential

The particle size, PDI, and zeta potential of nano-WalPI, nano-WPI, and nano-WalPI-WPI at pH 7.0 were determined using a Zetasizer Nano ZS (Malvern, UK). The dispersant was distilled water with a refractive index of 1.33.

#### 2.4.4. Measurement of H_0_

The H_0_ of nano-WalPI, nano-WPI, and nano-WalPI-WPI was determined according to the method of Wu et al. [32] with slight modifications. Protein solutions with concentrations of 0.02%, 0.04%, 0.06%, 0.08%, and 0.1% (*w*/*v*) were prepared using phosphate buffer (pH 7.0, 0.01 mol/L), and 4 mL was taken and mixed with 20 μL (8-anilino-1-naphthalene sulfonic acid) ANS (8.0 mol/L) and allowed to react for 15 min in the dark. The parameters of the enzyme analyzer (MD SpectraMax Plus384, BIOTEK, Winooski, VT, USA) were set with an excitation wavelength of 390 nm, an emission wavelength of 484 nm, and a slit width of 2 nm. A linear regression was performed with protein concentration as the x-axis and fluorescence intensity as the y-axis, and the slope was defined as H_0_.

#### 2.4.5. Thermal Stability

Differential scanning calorimetry (DSC) (3500 Sirius, Netzsch, Germany) was used to analyze the thermal stability of nano-WalPI, nano-WPI, and nano-WalPI-WPI. The heating rate was 10 °C/min, starting from 25 °C and reaching a final temperature of 180 °C, with a nitrogen flow rate of 20 mL/min.

#### 2.4.6. Infrared Spectroscopy Analysis

The infrared spectra of nano-WalPI, nano-WPI, and nano-WalPI-WPI were determined using a Fourier transform infrared (FTIR) spectrometer (650, Tianjin, China). The scanning range was from 400 to 4000 cm^−1^ with 16 scans and a resolution of 4 cm^−1^.

#### 2.4.7. Endogenous Fluorescence Spectroscopy

Based on the combined analysis of particle size, PDI, zeta potential, and H_0_, WalPI-WPI 7:3 demonstrated the optimal overall performance and was thus selected for this part of the experiment. The endogenous fluorescence spectroscopy method of He et al. [33] was followed with slight modifications. To prepare the protein solution, WalPI and WPI were mixed in a 7:3 (*w*/*w*) ratio to achieve a final concentration of 1.0% (*w*/*v*). The pH of the solution was adjusted to 12.0 using NaOH (2.0 mol/L), followed by stirring at 500 rpm for 4 h at room temperature. Subsequently, 0.1 mol/L of sodium dodecyl sulfate (SDS), thiourea, and NaCl were each added to the sample to achieve a final concentration of 10.0 mmol/L. The pH of the solution was adjusted to 7.0 using HCl (2.0 mol/L). Endogenous fluorescence spectra were obtained at an excitation wavelength of 280 nm and emission wavelengths of 400–450 nm, with a slit width of 2 nm.

#### 2.4.8. Exogenous Fluorescence Spectroscopy

The exogenous fluorescence spectroscopy method of Zhu et al. [34] was followed with slight modifications. The mixed solution (0.1%, *w*/*v*) of WalPI and WPI (WalPI to WPI ratios of 9:1, 8:2, 7:3, 6:4, and 1:1, respectively) was adjusted to pH 12.0 using NaOH (2.0 mol/L) and stirred at 500 rpm for 4 h at room temperature. Next, the pH was adjusted to 12.0, 11.0, 10.0, 9.0, 8.0, and 7.0 using HCl (0.1 mol/L), respectively. Then, 4 mL of the solution was mixed with 20 μL of ANS (8.0 mol/L) under light-shielded conditions for 15 min. The exogenous fluorescence spectroscopy intensity was measured at an excitation wavelength of 390 nm and an emission wavelength of 484 nm.

#### 2.4.9. Emulsifying Properties

The emulsifying properties of nano-WalPI, nano-WPI, and nano-WalPI-WPI were determined based on the procedures of Liu et al. [35]. Corn oil was employed as the oil phase, while a protein solution (3.0%, *w*/*v*) served as the aqueous phase. The oil phase (75%, *v*/*v*) and the aqueous phase were homogenized at 15,000 rpm for 5 min using a high-speed disperser (FJ200-SH, Wuxi, China). Subsequently, 100 μL samples of the emulsions were extracted at 0 min and 30 min from a point 0.5 cm above the bottom of the centrifuge tube and diluted at a ratio of 1:300 with 0.1% (*w*/*v*) SDS. Finally, the absorbance at 500 nm of the diluted emulsion was measured using a UV-visible spectrophotometer (UV-2600, Shimadzu, Japan). The emulsifying activity index (EAI) and emulsifying stability index (ESI) were calculated according to Formulas (2) and (3).

(2) $${\mathrm{EAI}\;(m}^{2}/g)=2\times 2.303\times \frac{A_{0}\times \;N}{C\times \varphi \times L\times 10000}$$

(3) $$\mathrm{ESI}\;(\min)=\frac{A_{0}}{A_{0}-A_{30}}\times 30$$

where A_0_ is the absorbance at time 0 min, N is the dilution factor (300), C is the protein concentration (0.03 g/mL), φ is the oil volume fraction (0.75), L is the optical path (1 cm), and A_0_ is the absorbance at time 30 min.

### 2.5. Preparation and Characteristics of HIPEs

#### 2.5.1. Preparation of HIPEs

HIPEs of nano-WalPI, nano-WPI, and nano-WalPI-WPI were prepared according to the method of Zhao et al. [36] with slight modifications. The protein solution (3.0%, *w*/*v*) was stirred at 500 rpm for 4 h at room temperature and subsequently hydrated at 4 °C overnight. The solution was then homogenized using a high-speed disperser at 15,000 rpm for 5 min, during which corn oil was gradually introduced. The oil phase constituted 75% (*v*/*v*) to form HIPEs.

#### 2.5.2. Droplet Size

The droplet size of HIPEs was measured using a laser scattering particle size distribution analyzer (LA-960V2, Horiba, Kyoto, Japan). The temperature was 25 °C. Distilled water was used as the dispersing medium with a refractive index of 1.33. The software provided by the instrument vendor analyzed the data, which was represented as the average droplet size.

#### 2.5.3. Microscopic Morphology

The microscopic morphology of HIPEs was observed using an optical microscope (SK2009, Saike Digital Technology Co., Ltd., Shenzhen, China) at 400× magnification.

#### 2.5.4. Macroscopic Observation

Macroscopic observation was performed on fresh HIPEs and HIPEs that had been stored at room temperature for 15 days. Additionally, the emulsion states were observed and photographed on days 1, 5, 9, and 15.

#### 2.5.5. Rheological Properties

The rheological behavior of the HIPEs was measured using a rheometer (HR 20 Discovery, TA Instruments, New Castle, DE, USA). The parallel plate geometry with a diameter of 40 mm was employed, with the measurement gap and temperature set to 1 mm and 25 °C, respectively. Viscosity was evaluated by varying the shear rate from 0.1 to 100 s^−1^, and the corresponding changes in viscosity were recorded. Frequency scans ranging from 0.1 to 100 Hz (with an oscillatory stress of 1 Pa) and stress scans ranging from 0.1 to 100 Pa (with an oscillatory frequency of 1 Hz) were performed to determine the storage modulus (G′) and loss modulus (G″) of the HIPEs. The thixotropic behavior of the HIPEs was assessed by altering the shear rate (0.1 s^−1^ and 10 s^−1^) and subsequently documenting the relationship between time and viscosity.

### 2.6. Statistical Analysis

All experiments were replicated at least three times. Statistically significant differences were determined using SPSS (version 26.0, SPSS Inc., Chicago, IL, USA). Differences were considered significant at *p* < 0.05 and were denoted by different lowercase letters (a–f).

## 3. Results and Discussion

### 3.1. Characteristics of Nano-WalPI-WPI

#### 3.1.1. SDS-PAGE

**Figure 1:**
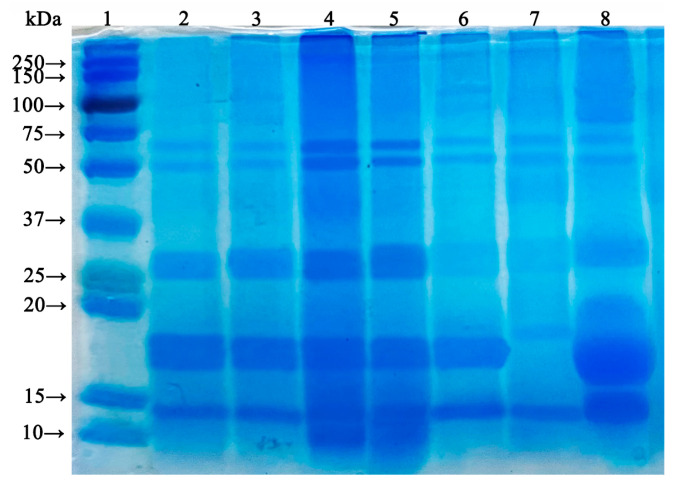
SDS-PAGE images of marker (lane 1), supernatant of WalPI-WPI 9:1 (lane 2), WalPI-WPI 8:2 (lane 3), WalPI-WPI 7:3 (lane 4), WalPI-WPI 6:4 (lane 5), WalPI-WPI 1:1 (lane 6), nano-WalPI (lane 7), and nano-WPI (lane 8). WalPI: walnut protein isolate; WPI: whey protein isolate; nano-WalPI: WalPI nanoparticles; nano-WPI: WPI nanoparticles; nano-WalPI-WPI: WalPI-WPI nanoparticles.

The supernatants of nano-WalPI, nano-WPI, and nano-WalPI-WPI were separately analyzed by SDS-PAGE. The SDS-PAGE images (Figure 1) showed that the subunit bands of the nano-WalPI-WPI (lane 2–6), nano-WalPI (lane 7), and nano-WPI (lane 8) ranged from 10 to 75 kDa. The molecular weight of gluten and albumen, which were the two main subunits in nano-WalPI, ranged from 10 to 37 kDa and 10 to 75 kDa, respectively [37]. The subunits of nano-WPI primarily consisted of bovine serum albumin (BSA), β-lactoglobulin (β-LG), and α-lactalbumin (α-LA), with molecular weights of 66.2, 18.4, and 14.2 kDa, respectively [38]. The SDS-PAGE images indicated that as the ratio of WPI increased, more β-LG integrated into the WalPI, resulting in a more pronounced coloration of the bands while retaining the intact primary structure of WalPI and WPI [39]. Thus, the pH cycling technique was effective in maintaining protein molecules.

#### 3.1.2. Solubility

**Figure 2:**
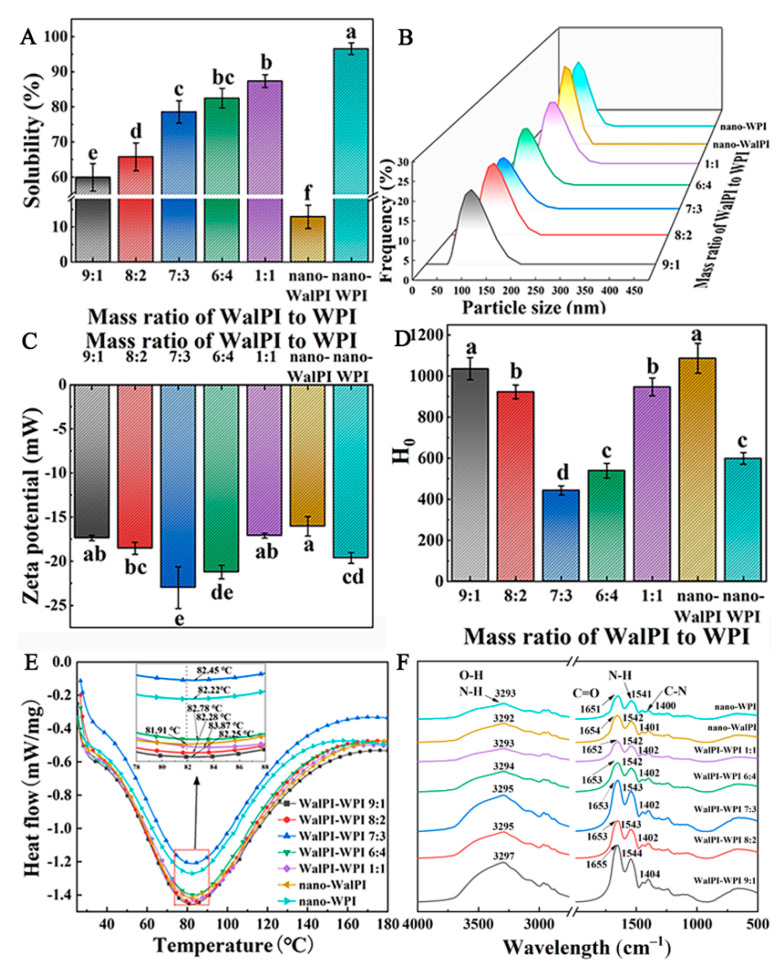
Solubility (**A**), particle size distribution (**B**), zeta potential (**C**), H_0_ (**D**), thermal stability (**E**), and FTIR spectra (**F**) of nano-WalPI, nano-WPI, and nano-WalPI-WPI. Different lowercase letters (a–f) indicate a significant difference (*p* < 0.05) between samples. H_0_: surface hydrophobicity; FTIR: fourier transform infrared.

As shown in Figure 2A, nano-WalPI had a solubility of less than 20%, while the solubility of nano-WPI was about 96.56%. As the ratio of WPI increased, the solubility of the nano-WalPI-WPI exhibited an increasing trend. The solubility of WalPI-WPI 1:1 was approximately 6.77 times that of nano-WalPI. This increase was due to the combination of WalPI with WPI, forming a higher-order structure and altering the original structural pattern of WalPI [40]. The solubility results indicated that WPI contributed to the improvement in WalPI solubility, and the pH cycling technique proved feasible for enhancing WalPI solubility [41].

#### 3.1.3. Particle Size and PDI

As shown in Table 1, for nano-WalPI, nano-WPI, and nano-WalPI-WPI, the average particle size was below 150 nm, and the PDI was under 0.4, signifying that a nano-sized particle was of an average size and exhibited a uniform distribution. The average particle sizes of nano-WalPI and nano-WPI were 146.77 nm and 107.87 nm, respectively. The average particle sizes of the nano-WalPI-WPI ranged from 70.98 to 124.57 nm. The average particle size of nano-WalPI-WPI was smaller than that of nano-WalPI, which was attributed to the enhanced hydrophobic interactions between WalPI and WPI during pH cycling acidification, resulting in a more compact structure. Zhan et al. [42] prepared WPI-zein composite nanoparticles by a pH cycling technique and encapsulated curcumin and similar results were found in the study of particle size. The particle size distribution chart (Figure 2B) showed a narrow peak width of nano-WalPI, nano-WPI, and nano-WalPI-WPI, further demonstrating the concentration of the particle size distribution. The average particle size of nano-WalPI-WPI initially decreased and then increased with the increase in the ratio of WPI, and there was no significant difference in PDI (*p* > 0.05). The enhanced interactions of WalPI with WPI resulted in a gradual decrease in the average particle size of nano-WalPI-WPI (WalPI-WPI 9:1 to WalPI-WPI 7:3). The average particle size and PDI of WalPI-WPI 7:3 were 70.98 nm and 0.207, respectively. Further increasing the ratio of WPI led to an increase in the average particle size of nano-WalPI-WPI to 124.57 nm, attributed to the fact that the binding sites of WalPI and WPI were close to saturation and unbound WPI were distributed on the surface of WalPI.

#### 3.1.4. Surface Properties

H_0_ and surface charge play an important role in maintaining the stability of a protein solution system, so the H_0_ and zeta potential of nano-WalPI, nano-WPI, and nano-WalPI-WPI were determined to characterize their surface properties. Zeta potential can assess the stability of nanoparticles in suspension: the larger the absolute value of the zeta potential, the more stable the system [43]. The ANS fluorescent probe can specifically bind to the hydrophobic regions of proteins, allowing for the detection of the number of hydrophobic groups present on the surface of protein molecules, indicated as H_0_ [44,45]. The absolute value of the zeta potential of nano-WalPI was smaller than that of nano-WPI, and the opposite was true for H_0_ (Figure 2C,D). After combining with WPI, the absolute value of zeta potential for nano-WalPI-WPI increased, and H_0_ decreased, indicating the enhancement in the surface charge and the generation of strong electrostatic repulsion, which were conducive to the improvement in its dispersibility in water; meanwhile, the hydrophobicity decreased. The alkalinization process in the pH cycle unfolded the WalPI and WPI structures and exposed the charged groups, leading to an increase in the absolute value of the zeta potential of nano-WalPI-WPI. The decrease in H_0_ was caused by the burial of hydrophobic groups after the pH was adjusted to neutral. This reduced the aggregation between nanoparticles and improved their stability in water. As the ratio of WPI increased, the absolute value of the zeta potential of nano-WalPI-WPI increased and then decreased, while H_0_ decreased and then increased, which was consistent with the trend of particle size changes. WalPI-WPI 7:3 had a high negative surface charge and low H_0_, indicating that WalPI-WPI 7:3 exhibited excellent surface properties in the system. This was conducive to the improvement in interfacial strength and interfacial stability. Further increasing the ratio of WPI, the absolute decrease in zeta potential and increase in H_0_ for nano-WalPI-WPI was because the WalPI and WPI binding sites were close to saturation, and the excess WPI was distributed on the WalPI surface, decreasing the exposure of negatively charged groups and the burial of hydrophobic groups.

#### 3.1.5. Analysis of Thermal Stability

DSC can be used to reflect the conformational stability of proteins by directly measuring the thermal changes that occur as the temperature of proteins increases or decreases [46]. The thermal stability of nano-WalPI, nano-WPI, and nano-WalPI-WPI is shown in Figure 2E. The denaturation peak temperature (T_d_) represents the critical temperature at which a protein transitions from its native state to a denatured state, and a higher value indicates a greater thermal stability of the protein [10]. The T_d_ of nano-WalPI and nano-WPI appeared at 81.91 °C and 82.22 °C, respectively. Compared to nano-WalPI, the T_d_ of WalPI-WPI 9:1 to WalPI-WPI 1:1 successively increased by 0.34 °C, 0.37 °C, 0.54 °C, 0.87 °C, and 1.96 °C, respectively, indicating that WPI was favorable to improve the thermal stability of WalPI and that the thermal stability of nano-WalPI-WPI increased with the increase in the ratio of WPI. This was attributed to the fact that β-LG, the major subunit in nano-WPI (about 65%) which was shown in Figure 1, began to denature above 70 °C, releasing sulfhydryl groups, which were converted to disulfide bonds [47]. Disulfide bonds play a role in stabilizing the spatial structure of protein and increasing the overall rigidity of protein. The incorporation of WPI made the spatial structure of nano-WalPI-WPI more rigid and exhibited higher thermal stability.

#### 3.1.6. Analysis of FTIR

The FTIR spectra of nano-WalPI, nano-WPI, and nano-WalPI-WPI are shown in Figure 2F. The peaks at 3297–3292 cm^−1^ were attributed to the overlap of O-H and N-H stretching vibrations [48]. The position of the nano-WalPI-WPI peak was slightly shifted compared to nano-WalPI and nano-WPI, suggesting that the binding of WalPI to WPI affects intermolecular hydrogen bonding [49]. At 1654 cm^−1^ and 1651 cm^−1^, nano-WalPI and nano-WPI exhibit absorption peaks, which are attributed to the stretching vibrations of C=O in the amide I band [50]. The peaks at 1542 cm^−1^ and 1402 cm^−1^ corresponded to the N-H bending vibration in the amide II region and the C-N stretching vibration in the amide III region, respectively [51]. The shifting of the characteristic peaks of nano-WalPI-WPI in the amide I and amide II regions may be attributed to the change in the functional group structure due to the hydrophobic interactions between WalPI and WPI [27].

#### 3.1.7. Analysis of Endogenous Fluorescence Spectroscopy

**Figure 3:**
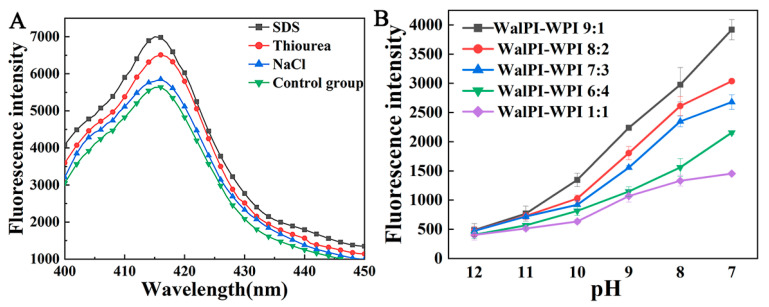
Fluorescence spectra of WalPI-WPI 7:3 with added blocking agents (**A**); ANS fluorescence spectra of nano-WalPI-WPI under varying pH (**B**). ANS: 8-anilino-1-naphthalene sulfonic acid.

SDS, thiourea, and NaCl possess the ability to disrupt hydrophobic interactions, hydrogen bonds, and electrostatic forces, respectively. Therefore, they can be used to investigate the interactions involved in the formation and stabilization of nano-WalPI-WPI [52]. Figure 3A shows that three blockers increased the fluorescence intensity of nano-WalPI-WPI, indicating that three types of non-covalent interactions played a facilitating role in nano-WalPI-WPI. Furthermore, the effect of SDS was the most pronounced, demonstrating that hydrophobic interactions are the primary driving force for the binding of WalPI and WPI.

#### 3.1.8. Analysis of Exogenous Fluorescence Spectroscopy

As shown in Figure 3B, the fluorescence intensity of nano-WalPI-WPI was lowest at pH 12.0. As the pH decreased from 12.0 to 7.0, the fluorescence intensity increased. This might be due to the increased interactions between WalPI and WPI, which enlarged the total surface area accessible to ANS, creating more binding sites for ANS [53]. The fluorescence intensity of nano-WalPI-WPI was enhanced with the pH decreased, indicating that WalPI and WPI folded during pH cycling and their binding affinity was enhanced. During neutralization, the fluorescence intensity of WalPI-WPI 9:1 was consistently the greatest, demonstrating a higher degree of folding and greater exposure of hydrophobic regions for stronger binding to ANS. As the ratio of WPI increased, the folding rate of the nano-WalPI-WPI decreased, and its structure became resistant to acid-induced hydrophobic folding. This indicated that the greater the ratio of WPI in the nano-WalPI-WPI, the greater its structural strength and resistance to folding.

#### 3.1.9. Analysis of Emulsifying Properties

**Figure 4:**
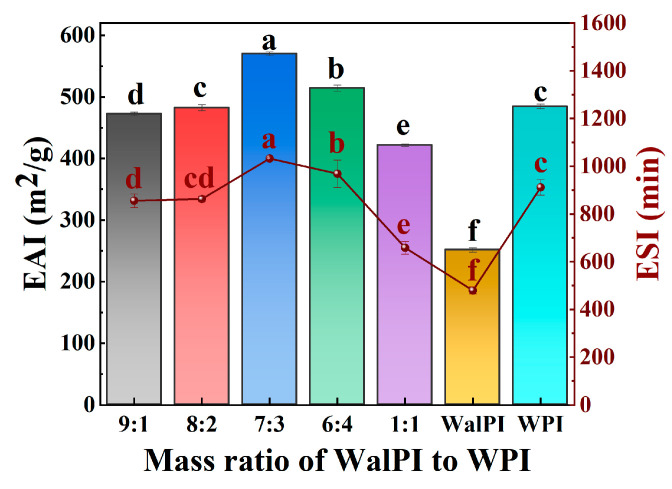
EAI and ESI of nano-WalPI, nano-WPI, and nano-WalPI-WPI. Different lowercase letters (a–f) indicate a significant difference (*p* < 0.05) between samples. EAI: emulsifying activity index; ESI: emulsifying stability index.

The capacity of proteins to stabilize the oil–water interface and adsorb quickly to the surface of oil droplets is usually indicated by their emulsifying properties [54], which were assessed by determining their ESI and EAI. Figure 4 shows the EAI and ESI of nano-WalPI, nano-WPI, and nano-WalPI-WPI. Compared with nano-WalPI with poor emulsifying properties (EAI and ESI of 251.59 m^2^/g and 479.27 min, respectively), nano-WalPI-WPI showed significant (*p* < 0.05) increases in both EAI and ESI, especially WalPI-WPI 7:3 with 570.53 m^2^/g and 1032.22 min, indicating that WPI had a positive effect on the emulsifying properties of WalPI. As the ratio of WPI increased, the EAI and ESI of nano-WalPI-WPI showed a tendency to increase and then decrease, which was consistent with the zeta potential results. Under electrostatic action, nano-WalPI-WPI could enhance the electrostatic repulsion between the emulsion droplets and form a tighter interfacial layer at the oil–water interface, thus improving the stability of the emulsion. Similar results were found in the emulsifying properties studies of whey protein concentrate–quercetin covalent and non-covalent complexes by Cheng et al. [55]. Meanwhile, smaller particles had a stronger adsorption capacity on the oil–water interface, which was conducive to the stability of the emulsion [56].

### 3.2. Properties of HIPEs

#### 3.2.1. Droplet Size

**Figure 5:**
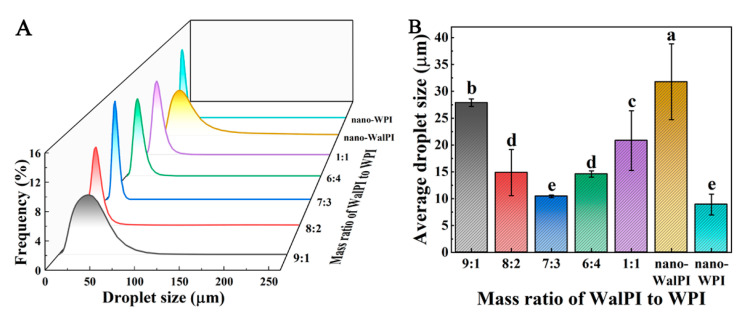
Droplet size distribution (**A**) and average droplet size (**B**) of HIPEs prepared with nano-WalPI, nano-WPI, and nano-WalPI-WPI. Different lowercase letters (a–e) indicate a significant difference (*p* < 0.05) between samples. HIPEs: high internal phase Pickering emulsions.

The droplet size and droplet size distribution of emulsions are important indicators of their stability, with smaller-sized particles resisting emulsion droplet aggregation and improving stability [57]. The smaller the range of droplet size distribution, the more homogeneous and stable the emulsion is [58]. Figure 5A,B show droplet size distribution and average droplet size of HIPEs prepared with nano-WalPI, nano-WPI, and nano-WalPI-WPI. The average size of all emulsions was less than 35 μm, and the droplet size distribution was single-peaked, approximating a normal distribution. The droplet size distribution range of nano-WalPI’s HIPEs was larger, and the droplet size was greater than that of the nano-WalPI-WPI in the emulsion. As the mass ratio of WPI increased, the range of the droplet size distribution and the average droplet size of nano-WalPI-WPI-stabilized HIPEs showed a tendency to decrease and then increase, as the difference in the HIPEs’ droplet size was related to the nanoparticle size. WalPI and WPI were tightly bound to form stable nano-WalPI-WPI through interactions (hydrophobic interactions, hydrogen bonds, and electrostatic forces), which adsorbed at the oil–water interface to form a dense network, providing stabilization for the emulsions. Meng et al. [59] found that chitosan and polyphenol were tightly bound by hydrogen bonds and electrostatic interactions at the oil–water interfacial layer of Pickering emulsion, which was dense and stable to resist the effects of different external environmental factors. Chen et al. [60] also found that TA and ovalbumin bind via hydrogen bonding and hydrophobic interactions, and TA improves the wettability of ovalbumin, resulting in greater interfacial stability at the oil–water interface of HIPEs. The emulsion of WalPI-WPI 7:3 exhibited the smallest average size, at just 10.45 μm, and a more concentrated droplet size distribution. This was due to the sufficient adsorption of the smaller particle size nano-WalPI-WPI at the oil–water interface, forming an interfacial layer that effectively prevents the coalescence of emulsion droplets, resulting in optimal emulsion stability.

#### 3.2.2. Microstructure

**Figure 6:**
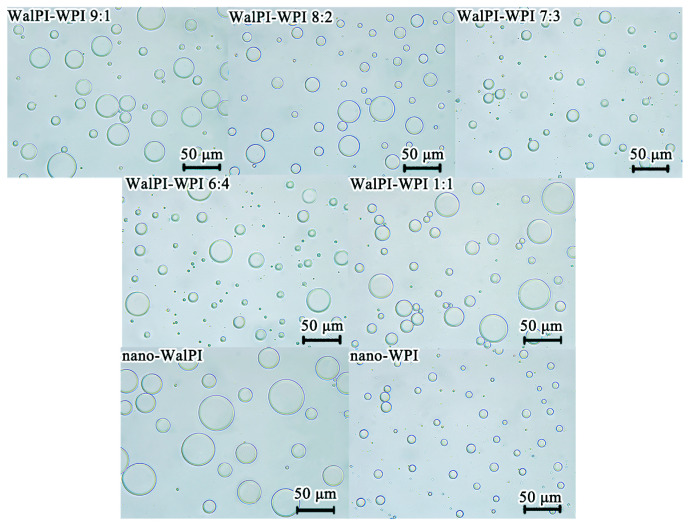
Microstructure of HIPEs prepared with nano-WalPI, nano-WPI, and nano-WalPI-WPI.

The microstructure of HIPEs (Figure 6) showed that all emulsion droplets were spherical, structurally intact, and uniformly dispersed. The droplets of nano-WalPI-stabilized HIPEs were not uniform, with a higher ratio of larger droplets. This was attributed to the lower surface charge of nano-WalPI, resulting in less electrostatic repulsion at the oil–water interface, which led to the aggregation of the droplets. The droplet sizes of HIPEs from nano-WalPI-WPI were reduced compared to nano-WalPI, with WalPI-WPI 7:3 having the smallest and most uniformly distributed droplet size. This was consistent with the results of the composite nanoparticle size study in Section 3.1.3. The droplet size difference proved that nano-WalPI-WPI, with their smaller sizes and hydrophilicity, resulted in smaller droplet sizes for HIPEs. Nanoparticles with small particle sizes had a large specific surface area, which was conducive to the adsorption of proteins at the oil–water interface, improving emulsification and forming a stable emulsion system [61].

#### 3.2.3. Macroscopic Observation

**Figure 7:**
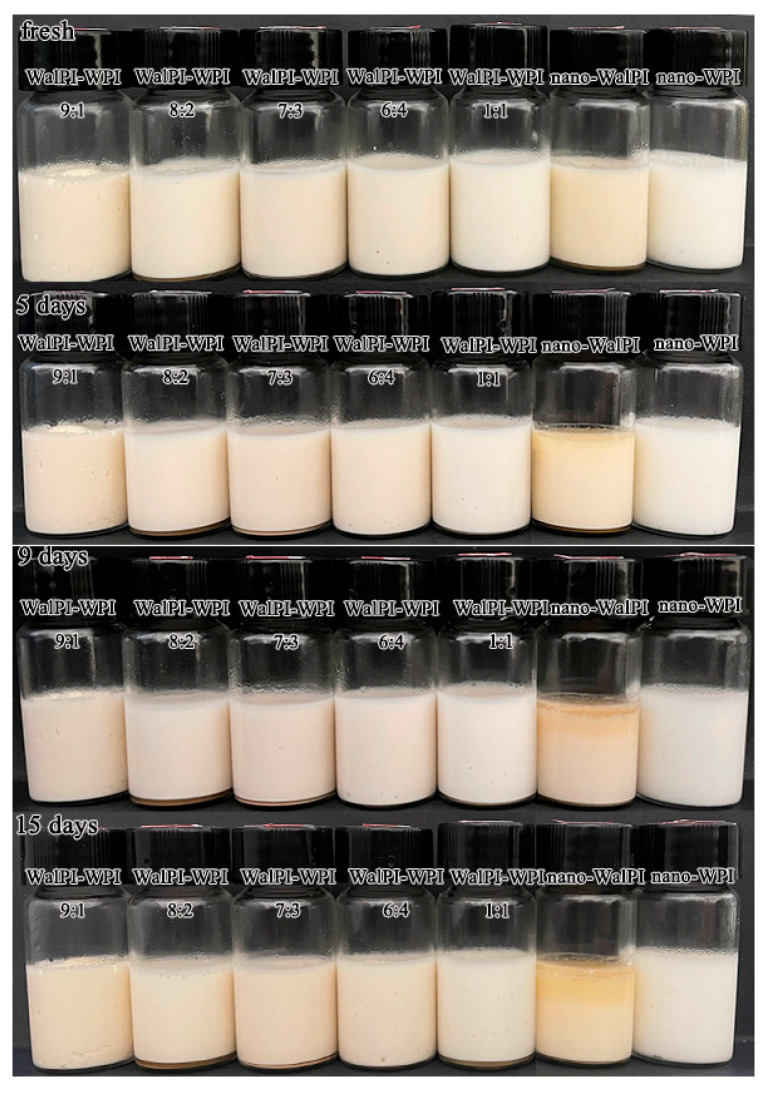
Storage stability of HIPEs prepared with nano-WalPI, nano-WPI, and nano-WalPI-WPI.

The appearance of the HIPEs stored for 15 days is shown in Figure 7. HIPEs stabilized by nano-WPI and nano-WalPI-WPI were milky white, while the nano-WalPI emulsion was yellowish because of the color of WalPI. The freshly prepared HIPEs were stable, with no differences observed among the samples. The HIPEs of nano-WalPI began to stratify on the fifth day, showing the worst stability due to the larger particle size, and the lower surface charge of nano-WalPI, which led to weaker emulsification capability. As a result, the formed emulsion had larger droplets, rendering the emulsion system unstable. The phase separation of HIPEs of nano-WalPI tended to increase with storage time and was most pronounced after the 15th day. The HIPEs stabilized by nano-WalPI-WPI were homogeneous during the storage period (1–15 days) because the small-size particles and high surface charge could provide sufficient spatial potential resistance and electrostatic repulsion to form a tight interfacial adsorbent layer and a network structure of the emulsion droplets, which could inhibit the emulsion from agglomerating and flocculating during storage [62].

#### 3.2.4. Rheological Behavior

**Figure 8:**
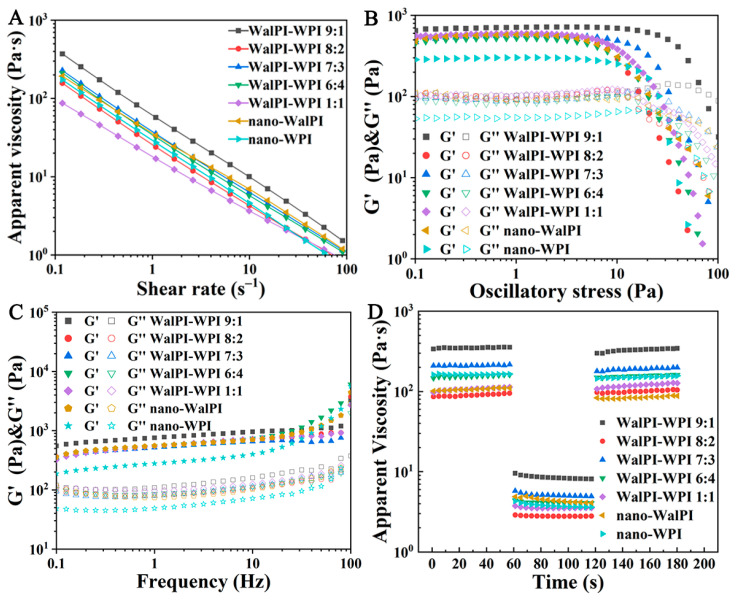
Rheological properties of protein HIPEs prepared with nano-WalPI, nano-WPI, and nano-WalPI-WPI. Logarithmic plot of emulsion viscosity (**A**); G′ and G″ of the emulsion during stress scanning (**B**); G′ and G″ of the emulsion during frequency scanning (**C**); Thixotropic diagram of emulsion (**D**).

As shown in Figure 8A, the viscosity of all HIPEs decreased with increasing shear rate, exhibiting typical shear-thinning behavior and showing pseudoplastic fluid rheological properties [63]. The reason is that as the shear rate increases, sufficient stress causes the rate of internal structure breakdown within the droplets to exceed the rate of reconstruction, leading to a reduction in the flow resistance between droplets and a decrease in viscosity [64]. At the same shear rate, the HIPEs of WalPI-WPI 1:1 had the lowest viscosity, and the HIPEs of WalPI-WPI 9:1 had more pronounced pseudoplastic characteristics, followed by WalPI-WPI 7:3.

Oscillatory testing (stress and frequency sweeps) yielded G′ and G″, reflecting the elastic and viscous magnitude. The stress results (Figure 8B) showed that G′ was larger than G″, and the modulus remained stable in the low-stress range (0.1–10 Pa), indicating that HIPEs stabilized by nano-WalPI, nano-WPI, and nano-WalPI-WPI were predominantly elastic with a gel-like structure. Nanoparticles can hinder the movement and aggregation of droplets, increasing the possibility of long-term stable storage [65]. Differences in the strength of the gel network of different protein emulsions were caused by various factors such as the flocculation of emulsion droplets, rearrangement of protein structures at the oil–water interface, and interactions between protein molecules [66]. Under the high-stress range (10–100 Pa), G′ and G″ showed a decreasing trend, with the emergence of a crossover point, indicating the disruption of the network structure of the emulsion.

The variation in HIPEs’ G′ and G″ with shear frequency is shown in Figure 8C. G′ was always higher than G″ for all HIPEs throughout the frequency range. Additionally, the weak frequency dependence of G′ and G″ in the low-frequency range (0.1–10 Hz) suggested that the HIPEs were more stable and had a typical highly flocculated elastic structure [67]. In the high-frequency range (10–100 Hz), the G′ of WalPI-WPI 6:4-, nano-WalPI- and nano-WPI-stabilized HIPEs increased significantly with frequency, indicating that they were not sufficiently stabilized. In contrast, the G′ of WalPI-WPI 7:3-stabilized HIPEs did not change significantly, showing excellent structural stability. There was no significant difference in the trend of G″ with frequency for different samples.

Thixotropic experiments were conducted to explore the degree of recovery of the HIPEs’ structure under external forces. After the shear treatment of low rate (0.1 s^−1^)–high rate (10 s^−1^)–low rate (0.1 s^−1^), the change in HIPEs’ viscosity with time is shown in Figure 8D, presenting three stages. When the shear rate increased to 10 s^−1^, the structural strength of the HIPEs weakened and the viscosity decreased immediately. When the shear rate returned to 0.1 s^−1^, the HIPEs’ viscosity experienced a rebound process and returned to the original state, which was only slightly lower than in the first stage. This occurred because the HIPE droplets underwent directional flow at a high shear rate and returned to a disordered state at a low shear rate [68]. This showed that the process of structural destruction was reversible when HIPEs were subjected to shear. There was no significant difference in the degree of structural recovery of HIPEs stabilized by nano-WalPI, nano-WPI, and nano-WalPI-WPI, all of which possessed good thixotropic properties.

## 4. Conclusions

In this study, nano WalPI-WPI was synthesized using a pH cycling technique and was found to effectively stabilize HIPEs. The integration of WPI significantly enhanced the functional properties of WalPI, including solubility, thermal stability, hydrophilicity, and emulsifying properties. The synergistic interaction between WalPI and WPI, mediated through hydrophobic interactions, hydrogen bonding, and electrostatic forces, maintained the primary structure of WalPI and WPI, increasing its resistance to acid-induced denaturation. Compared with nano-WalPI, HIPEs prepared by nano-WalPI-WPI had smaller droplet sizes and showed high stability in 15 days of storage without significant phase separation. The HIPEs stabilized by nano-WalPI-WPI exhibited a microstructure of intact spherical droplets, forming an elasticity-dominated gel-like structure with favorable thixotropic properties. Among all the samples, the WalPI-WPI 7:3 and its corresponding HIPEs demonstrated the best overall performance. This study provided a proven technical method for the application of hydrophobic proteins and protein-based HIPEs in the food field.

## Figures and Tables

**Table 1:** Average particle size and PDI of nano-WalPI, nano-WPI, and nano-WalPI-WPI. PDI: polydispersity index.

Samples	Average Particle Size (nm)	PDI
WalPI-WPI 9:1	108.80 ± 12.565 ^b^	0.316 ± 0.100 ^ab^
WalPI-WPI 8:2	103.23 ± 4.389 ^b^	0.326 ± 0.095 ^ab^
WalPI-WPI 7:3	70.98 ± 0.779 ^c^	0.207 ± 0.013 ^ab^
WalPI-WPI 6:4	75.71 ± 1.038 ^c^	0.193 ± 0.054 ^b^
WalPI-WPI 1:1	124.57 ± 4.917 ^ab^	0.295 ± 0.046 ^ab^
nano-WalPI	146.77 ± 27.943 ^a^	0.341 ± 0.152 ^a^
nano-WPI	107.87 ± 5.220 ^b^	0.270 ± 0.038 ^ab^

Different lowercase letters (a–c) indicate a significant difference (*p* < 0.05) between samples.

## Data Availability

The original contributions presented in the study are included in the article, further inquiries can be directed to the corresponding author.
